# Psychometric properties of the Psychological Consequences of Screening Questionnaire (PCQ) adapted for cognitive screening

**DOI:** 10.1002/alz.090010

**Published:** 2025-01-09

**Authors:** Rebecca Lovett, Sarah Filec, Jeimmy Hurtado, Mary Kwasny, Alissa Bernstein Sideman, Stephen Persell, Katherine L Possin, Michael S Wolf

**Affiliations:** ^1^ Northwestern University, Chicago, IL USA; ^2^ Global Brain Health Institute, San Francisco, CA USA; ^3^ University of California, San Francisco, San Francisco, CA USA; ^4^ Memory and Aging Center, UCSF Weill Institute for Neurosciences, University of California, San Francisco, San Francisco, CA USA; ^5^ Northwestern University Feinberg School of Medicine, Chicago, IL USA

## Abstract

**Background:**

Context‐specific measures with high content validity are needed to adequately determine psychosocial effects related to screening for cognitive impairment. The objective of this investigation was to examine psychometric properties of the Psychological Consequences of Screening Questionnaire (PCQ), a measure of psychological impact of medical screening, adapted for cognitive screening in primary care.

**Methods:**

Two‐hundred adults aged ≥65 recently completing routine, standardized cognitive screening as part of their Medicare Annual Wellness Visit were administered the adapted PCQ measure, comprised of negative (PCQ‐Neg) and positive (PCQ‐Pos) scales. Measure distribution, acceptability, internal consistency, factor structure, and content validity (construct, discriminative, criterion) were analyzed.

**Results:**

Participants had a mean age of 73.3, were primarily female and socioeconomically advantaged. Nearly all received a normal cognitive screening result (99.5%). Overall PCQ scores were low (PCQ‐Neg: M 1.27; PCQ‐Pos: M 7.63). Both scales demonstrated floor effects. Acceptability was satisfactory, although the PCQ‐Pos had slightly more item missingness. Both scales had Cronbach alpha’s >0.80 and a single factor structure. Spearman correlations between the PCQ‐Neg with general measures of psychological distress ranged from 0.26 to 0.37 (p’s <0.001); correlation with a measure of well‐being was ‐0.19 (p<0.01). The PCQ‐Neg discriminated between those with and without a subjective cognitive complaint (M 2.73 vs. 0.89, p <0.001), and was associated with medical visit satisfaction (r = ‐0.24, p<0.001). The PCQ‐Pos predicted willingness for future screening (M 8.00 vs 3.00, p = 0.03).

**Conclusions:**

The adapted PCQ‐Neg scale is an overall valid measure of negative psychological consequences of cognitive screening; findings for the PCQ‐Pos scale were more variable. Future studies are needed to further understand PCQ performance among more diverse samples and those with abnormal test results following cognitive screening.

